# Application of *Sporosarcina pasteurii* for the biomineralization of calcite in the treatment of waste concrete fines

**DOI:** 10.1007/s11356-025-36102-2

**Published:** 2025-02-26

**Authors:** Kristyna Klikova, Petr Holecek, Vaclav Nezerka, Zdenek Prosek, Dana Konakova, Katerina Demnerova, Hana Stiborova

**Affiliations:** 1 Faculty of Food and Biochemical Technology, University of Chemistry and Technology, Technicka 3, 166 28 Prague 6, Czech Republic; 2 Faculty of Civil Engineering, Czech Technical University in Prague, Thakurova 2077/7, 166 29 Prague 6, Czech Republic

**Keywords:** MICP, *Sporosarcina pasteurii*, Ureolytic activity, CaCO_3_ crystals, Waste concrete fines

## Abstract

In this study, we explored and described various parameters of microbially induced calcite precipitation (MICP) using the alkaliphilic bacterium *Sporosarcina pasteurii* DSM 33, which exhibits ureolytic activity, to stabilize and strengthen waste concrete fines (WCF). Bacterial cell concentration, single and repeated addition of bacterial suspension, and pH adjustment were tested in stage 1 of the experimental agenda in order to tune parameters for sample preparation in stage 2 focused on the effect of MICP treatment duration (14, 30, 60, and 90 days). Two types of WCF materials differing in their physicochemical properties were used for the stabilization. The results of the EDS and XRD analyses confirmed the presence of CaCO_3_ crystals, which increased by about 10–12% over time, affecting the porosity, compactness, and strength of the formed composites. The XRD results also indicated that the WCF properties significantly influence the formation of the type of CaCO_3_ crystals, supported also by microscopy observations. This study highlights the potential of MICP technology to make concrete recycling more sustainable, aligning with the concept of a circular economy; however, the interplay between the WCF materials of various properties and bacterial activity must be further scrutinized.

**Supplementary information:**

The online version contains supplementary material available at 10.1007/s11356-025-36102-2.

## Introduction

With the adoption of the Paris Climate Agreement in 2015 (Fahimizadeh et al. 2020; Stern and Valero 2021) and the Green Deal for Europe in 2019, global society has committed to reducing greenhouse gas and carbon dioxide emissions (Stern and Valero 2021; Kludze et al. 2022). The overproduction of CO_2_, a primary driver of global warming, plays a major role in the greenhouse effect (Pandey and Kumar 2014; Ebi et al. 2021).

In this context, the construction sector emerges as a significant contributor to greenhouse gas emissions (Pandey and Kumar 2019), accounting for roughly 7% of global CO_2_ emissions (Fahimizadeh et al. 2020). Portland cement production, an essential binding component of most construction materials (Mohamad et al. 2022; Nodehi et al. 2022), accounts for 80% of these emissions (Dapurkar and Telang 2017; Adesina 2020; Li et al. 2022b), mainly due to extraction of natural resources (Mohamad et al. 2022), and high energy consumption (Huntzinger and Eatmon 2009). Other environmental impacts include noise pollution, the production of wastewater (Dunuweera & Rajapakse 2018), vegetation degradation, soil erosion, or biodiversity loss (Mohamad et al. 2022).

The efforts to reuse and recycle are also driven by legislation measures addressing the huge amount of waste generation, with the majority of it (45–65%) landfilled (Lima et al. 2021). The current recycling methods for waste concrete handling involves crushing the material with subsequent separation of fractions (Ding et al. 2022), yielding coarse and fine aggregates and fine powder (C. Sun et al. 2020). Coarse aggregates with a diameter greater than 4.75 mm (Luo et al. 2022) have a high potential for use in new concrete, road bases, or surface material, among other applications (Vanderzee and Zeman 2018; Sun et al. 2020; Ding et al. 2022; Nežerka et al. 2023a). However, recycled aggregates can have issues with workability, lower strength or durability, which can be resolved through consistent purification or carbonization processes (Prošek et al. 2020; C. Sun et al. 2020).

Waste concrete fines (WCF) particles with a diameter of less than 4.75 mm (Luo et al. 2022) account for up to 15–50% of the volume of all crushed concrete, but their use is often regulated and restricted by legislation (Evangelista et al. 2015; Prošek et al. 2020; Nežerka et al. 2023a). WCF has great potential for reuse, but storage and long-term preservation are challenges that often lead to landfilling of the material (Ding et al. 2022; H.-F. Li et al. 2022a). The effectiveness of using WCF in concrete largely depends on their particle size, which is typically in the micrometer range. However, smaller particle sizes are associated with lower density and higher water absorption (Rodrigues et al. 2013), which can negatively impact the long-term durability and strength of concrete (Xiao et al. 2018), and are significantly affected by contaminants due to the recycling process (Evangelista et al. 2015). Despite these drawbacks, small particle sizes offer residual reactivity, as investigated by Pawluczuk (2017) and Xiao et al. (2018). In a prior review (Nežerka et al. 2023a, b), we theorized the potential application of a biomineralization process, specifically microbially induced calcite precipitation (MICP), for cementing WCF. This approach has promise as a viable strategy to not only reduce the carbon footprint, but also embody the principles of a circular economy.

MICP is a natural biomineralization process that uses microorganisms capable of biologically synthesizing minerals via precipitation (Qabany et al. 2012; Castro-Alonso et al. 2019). During MICP, microorganisms throughout different metabolic processes such as urea hydrolysis, oxidation of organic acids, amino acid ammonification, or photosynthesis (Castanier et al. 1999), and specific enzymes, for example, urease (for the hydrolysis of urea to form ammonia) (Castro-Alonso et al. 2019) or carbonic anhydrase, convert a supplied substrate (chemical component) into precipitated minerals such as calcium carbonate (Dapurkar and Telang 2017). Most studies (Torres-Aravena et al. 2018; Ghosh et al. 2019; Naveed et al. 2020; Leeprasert et al. 2022; Nasser et al. 2022) have verified that urea hydrolysis stands out as the most efficient process in terms of easiness of process control, high substrate solubility and, importantly, rapid precipitation effectiveness. The precipitated calcite can act as a binder on surfaces, and this feature can be utilized in various applications, e.g., for crack healing and pore plugging for self-healing concrete (Abdel Gawwad et al. 2016; Pungrasmi et al. 2019; Sun et al. 2019b; Intarasoontron et al. 2021; Sun et al. 2021b), for the production of sandstone bricks (Sun et al. 2021a) or other different types of concrete materials (Intarasoontron et al. 2021; Skevi et al. 2021; Zhao et al. 2021a, 2021b), for soil improvements such as erosion control (Sun et al. 2022), soil consolidation and solidification (Sun et al. 2021a; Sun et al. 2022), and bioremediation of heavy metal-contaminated soil (S. Wang et al. 2023; W. Zhang et al. 2023). Recently, some researches also deal with enzyme-induced carbonate precipitation technology (EICP) as an alternative way for environment-friendly sand consolidation (Ahenkorah et al. 2021; H. Wang et al. 2022a).

This study examined various conditions to optimize the biocementation process for binding WCF particles to form compact composite samples. Biocementation using MICP has been conducted on various materials such as coarse aggregates (Mahawish et al. 2018; Nagy et al. 2023; Sun et al. 2024), sands (Liufu et al. 2023; Zeitouny et al. 2023; Zeitouny et al. 2023; Wang et al. 2024), or soil (Mujah et al. 2017; Bibi et al. 2018; Ji et al. 2024), but there has been no description of the recycling of WCF, focusing on the various factors that affect the compactness and strength of the samples. The biocementation process in this study was achieved using the alkaliphilic bacterium *Sporosarcina pasteurii* DSM 33 (with proven ureolytic activity), which facilitated the precipitation of calcium carbonate crystals in the presence of urea and calcium ions. We hypothesized that adding essential nutrients and substances will enable the *S. pasteurii* DSM 33 precipitation of calcite, consequently strengthening the WCF, and that the physicochemical properties of WCF could affect the final properties of the formed composite samples.

## Materials and methods

### WCF

For our study, WCF were obtained by grinding concrete of different types and ages. The first WCF material, WCF-G, was prepared from a newly manufactured vibro-compacted concrete gutter. This prefabricated product was designed for surface water drainage. As it was not intended for structural purposes and the production was carried out in a plant under controlled conditions, it contained a minimum amount of Portland cement, yielding a strength class of C12/15. As a result of the low cement-to-aggregate ratio, WCF-G contained a large amount of aggregate and virtually no unhydrated clinker (Prošek et al. 2020). The WCF-G powder was prepared by grinding a separated fraction of size < 0.25 mm obtained after the gutter concrete crushing.

The second WCF material, WCF-H, was prepared by grinding stripped concrete from the Czech D1 highway, which connects the cities of Prague and Brno, during its reconstruction. It was made from a 60-year-old concrete obtained by crushing and grinding the structural layers of the modernized D1 highway section between Loket and Hořice (km 66.32–75.92). The concrete used in the highway construction was classified according to the EN 260–1 standard, with strength grades ranging from C16/20 to C25/30, indicating compressive strengths between 16 and 25 MPa at 28 days. During the processing of WCF from the highway concrete (WCF-H), particles exceeding 10 mm were excluded. The remaining material was crushed using a hammer crusher and then finely ground to a fraction < 1 mm using a Lavaris SKD 600 high-speed electric mill (230 kW).

**Fig. 1:**
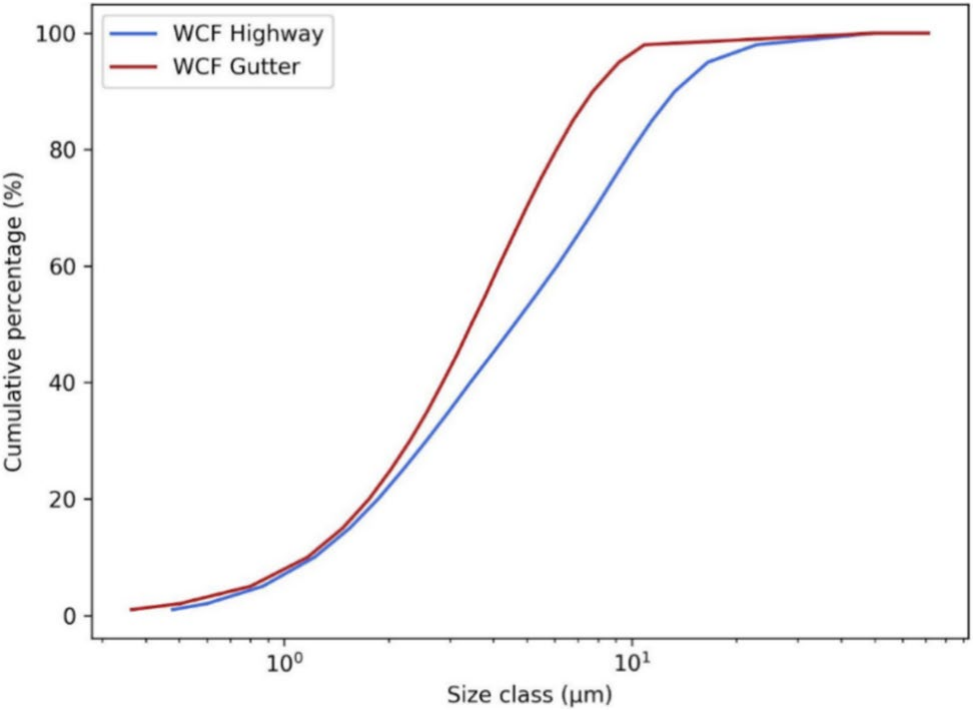
Cumulative particle-size distribution for the studied WCF materials determined using fritsch analyssete 22 micro tec plus laser diffraction particle size analyzer

As a result of the different grinding processes and aggregate composition within the recycled concrete samples, the particle size distribution of WCF materials was different. WCF-G samples contained smaller particles (mean diameter 5.08 µm), while the distribution of WCF-H was shifted towards larger fractions (mean diameter 7.33 µm). The particle size distribution curves are provided in Fig. 1.

### Bacterial strain, culture media, and growth conditions

This study utilized the gram-positive, alkaliphilic bacterium *Sporosarcina pasteurii* DSM 33 (ATCC 11859), which exhibits ureolytic activity and was obtained from the German Collection of Microorganisms and Cell Cultures GmbH (DSMZ, Braunschweig) (Ghosh et al. 2019). *S. pasteurii* DSM 33 was cultivated in Tryptone soya broth (TSB, Oxoid) medium with the pH adjusted to 7.3 ± 0.2 and sterilized by autoclaving at 121 °C for 15 min and supplemented with urea at a final concentration of 20 g/L. The cultivation was performed at 28 °C with shaking at 120 rpm for 48 h. The strain was stored at 4 °C on Tryptone soya agar (TSA, Oxoid) with the addition of urea (20 g/L) and was routinely subcultured at fresh medium every 2–3 weeks to maintain its viability. To induce the precipitation of calcite crystals and the binding of WCF particles, a biocementation solution consisting of Nutrient broth (NB, Oxoid) medium (4 g/L, pH adjusted to 6.8 ± 0.2), urea (20 g/L), and CaCl_2_ (2.8 g/L) was used. The stock solutions of urea (160 g/L) and CaCl_2_ (28 g/L) were always sterilized by filtering through PTFE filters (0.2-μm pore size, Sigma-Aldrich) and added into media after autoclaving.

### Ureolytic activity assay

Ureolytic activity was determined using the phenol-hypochlorite method described by G. Kim and Youn (2016). Briefly, *S. pasteurii* DSM 33 was grown in TSB supplemented with urea (20 g/L). The bacterial culture was centrifuged (6000*g*, 4 °C for 10 min), and the pellets were washed twice with 50 mM 4-(2-hydroxyethyl)piperazine-1-ethanesulfonic acid (HEPES) buffer. The cells were sonicated on ice (three pulses of 30 s each), and centrifuged (6000*g*, 4 °C for 10 min). The obtained supernatant (0.5 ml) was mixed with 0.5 ml of urease buffer (50 mM HEPES, 25 mM urea), and the resulting volume of 1 ml was incubated at 37 °C for 20 min. To stop the reaction, aliquots were transferred to 15-ml centrifuge tubes containing 1.5 ml of solution A (10 g/L phenol and 50 mg/L sodium nitroprusside, acting as a catalyst) and 1.5 ml of solution B (5 mg/ml NaOH and 0.044% v/v NaClO). The mixture was stirred and incubated at 37 °C for 30 min, and the absorbance was measured at a wavelength of 620 nm. Ureolytic activity was quantified in triplicate during the growth curve at time points of 0, 24, 38, 48, 65, 72, and 90 h. To construct a standard curve, ammonium chloride solution was utilized, as outlined in the protocol from the Urease Activity Assay Kit (ab204697, Abcam Trading Co., China): 0, 4, 12, 20, 32, 64, and 128 nmol/100 μl. Each concentration was transferred to a 96-well microtiter plate in 8 replicates, and the absorbance was measured using a spectrophotometer at a wavelength of 620 nm.

### Biocementation—experimental design

*S. pasteurii* DSM 33 was grown in TSB supplemented with urea (20 g/L) at 28 °C, 120 rpm. After 48 h, cells were centrifuged at 10 000*g* for 10 min at 4 °C, then washed and resuspended in a saline solution containing 9 g/L NaCl. The optical density at 600 nm (OD_600_) was adjusted by diluting the centrifuged suspension to 2.5 or 5.0 and further used for the MICP process. In stage 1, we adopted parameters from the literature, further discussed in the “Results and discussion” section, primarily to familiarize ourselves with MICP processes and to preliminarily confirm their applicability to WCF as opposed to soils or sands; rigorous testing and validation were reserved for stage 2, where we focused our main efforts to investigate the impact of WCF composition and MICP treatment duration.

### Optimization of MICP treatment (stage 1)

WCF suspensions for biocementation were prepared by mixing 7 g of WCF-G with 10 ml of sterile saline solution (9 g/L NaCl). During stage 1, we optimized preliminary parameters impacting sample compactness and integrity, as follows: (i) *S. pasteurii* DSM 33 bacterial cell concentration (OD_600_ values of 2.5 and 5.0), (ii) pH adjustment of the WCF suspension from an initial pH of 12.1–12.3 to 6.8 ± 0.2 using 1M HCl, and (iii) single or repeated applications of bacterial suspension (Table S1). Each variant was prepared in triplicate. For all samples, 10 ml of bacterial suspension was mixed with the WCF suspension and transferred to containers. After settling for 24 h, 10 ml of biocementation solution was added at 48-h intervals, alternating with the bacterial suspension in samples designated for repeated addition of bacterial suspension. Sterile (control) samples, which did not undergo MICP treatment, were prepared by mixing 7 g of sterile WCF-G (sterilized at 280 °C for 2 h) with saline solution added at corresponding time points instead of the biocementation solution. The temperature for MICP treatment was set up to 28 °C, which is optimal cultivation temperature for *S. pasteurii* DSM 33 (Okyay and Rodrigues 2014). After five additions of biocementation solution, all samples were dried, the compactness of the composite samples were analyzed.

The compactness of the biocemented WCF-G was recorded based on (i) the percentage of the largest associated material (> 15 mm) and consolidated grains of material (10–15 mm) and (ii) the frequency and thickness of cracks in biocemented conglomerates.

**Fig. 2:**
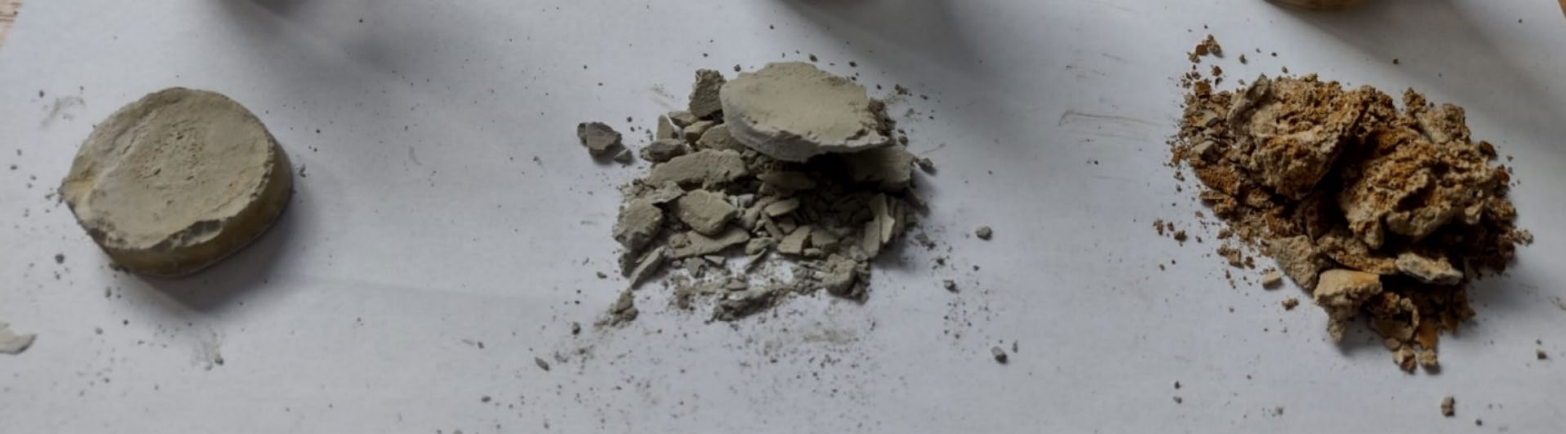
Illustration of sample compactness categories: non-cemented sample (right), partially cemented sample (middle), and fully cemented sample (left)

The samples were classified based on their compactness into three categories (Fig. 2):

1. Non-cemented: These samples had a powdery structure with no apparent cementation.
2. Disintegrated upon removal: These samples did not maintain structural integrity when taken out of the containers.
3. Stiff conglomerates: These stiff samples sustained handling.

Based on this classification, the parameters of the MICP treatment that showed the best compactness of the samples were selected for further experiments in stage 2.

### Effect of treatment duration (stage 2)

In stage 2, we further investigated the effects of MICP treatment duration together with impact of WCF properties (composition and particle mean size) on the strength and compactness of the resulting conglomerates. Therefore, within the stage 2, additional WCF material of differing origin and grading (WCF-H, as detailed in the “WCF” section) was used.

**Fig. 3:**
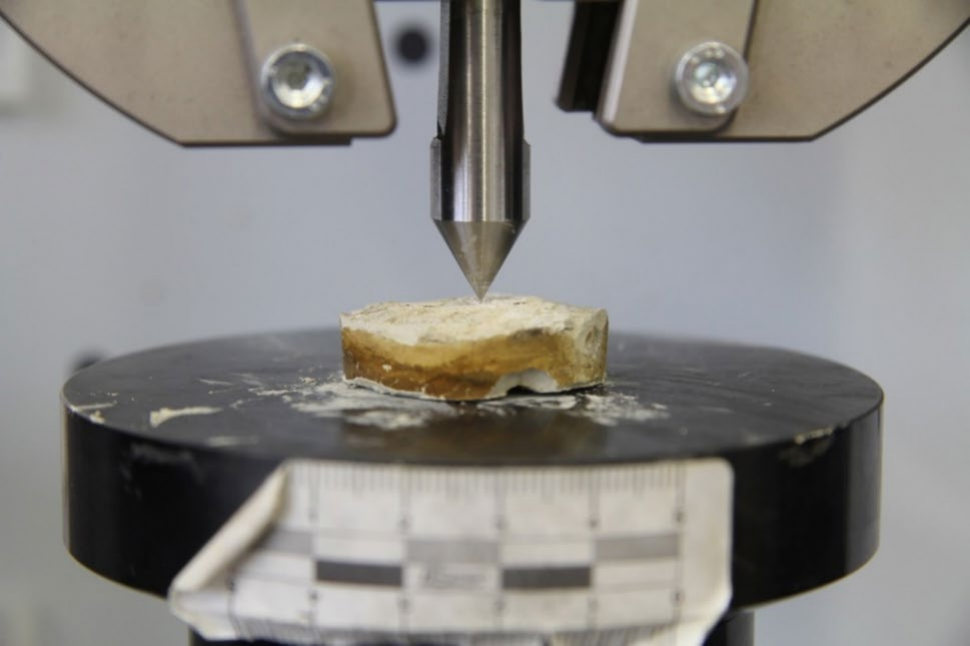
Indentation of hardened samples using a conically-shaped steel indenter

In stage 2, samples were prepared similarly to stage 1, with 7 g of WCF in 10 ml of saline solution, adjusting the pH to 6.8 ± 0.2 and using an *S. pasteurii* DSM 33 concentration of OD_600_ 5.0. Biocementation was carried out over various durations (14, 30, 60, and 90 days) to determine the optimal treatment period. The biocementation and saline solutions were added consistently every 48–72 h, with six replicates per sample (including sterile samples) (see Table S2). At the end of each treatment period, biocemented WCF samples were dried at 28 °C for 30 days before further analyses (SEM, XRD, and EDS). Compactness was evaluated visually, as desribed in stage 1, and strength of compact samples underwent macroscopic indentation testing (Fig. 3).

The macroscopic indentation (details and images provided in Holeček et al. (2023)) was conducted on selected samples using a displacement-controlled loading rate of 0.5 mm/min. The steel indenter was conically shaped, featuring a tip width of 0.15 mm, a base width of 12.2 mm, and a tip angle of 60°. The loading procedure was carried out with an MTS Criterion Series 40 loading frame.

### Scanning electron microscope: microscopy imaging

The calcium carbonate structures formed by bacteria or spontaneous carbonation were observed using a ZEISS FEG Merlin Scanning Electron Microscope (SEM) for microstructural and elemental analysis, equipped with a Schottky cathode (10 kV accelerating voltage, 1 nA current, 8.5 mm working distance, 50 μs dwell time per point, and 1024 px resolution). The vacuum required for SEM and sample preparation procedures kills bacteria, making it impossible to study their viability and the CaCO_3_ formation process.

Secondary electron (SE) detectors enabled the observation of the topology of the surfaces under investigation, while backscattered electron (BSE) detectors were employed to determine the distribution of phases in the samples based on the atomic numbers of their constituent elements. Elemental microanalysis was performed using an Oxford Instruments X-ray energy-dispersive spectrometer (EDS) to determine the mass and atomic percentages of chemical elements, both in point analysis and 2D fields. Phases were identified from their elemental compositions using stoichiometry.

Samples for SEM analysis were prepared in two forms: 

1. The samples were cut using a Struers Secotom 50 precision cutting machine to determine the distribution of individual phases and elements. Due to the brittle structure of the samples and to prevent disintegration and removal of CaCO_3_ during sample preparation, the samples were impregnated with Stuers EpoFix epoxy resin, which was subsequently pressed into the sample microstructure using a vacuum pump. Each batch and bacterial exposure time were represented by one sample. The prepared sample was then polished using SiC abrasive foils (grain size 1000–4000 grains/cm^2^). The sample was subsequently coated in an argon atmosphere with a 3-nm platinum layer to increase conductivity, which is essential for SEM and EDS analysis.
2. To determine the shape, size, and quantity of crystals, samples of the solidified material were ground again and placed on an adhesive target. These samples were also coated with a 6-nm-thick platinum layer in an argon atmosphere due to the increased number of irregularities on the sample.

### X-ray diffraction

Powder X-ray diffraction (XRD) was utilized to identify and assess individual phases of crystalline origin by recording the intensity of diffracted radiation when exposed to monochromatic X-ray radiation with gradually varying diffraction angles (Stanjek & Häusler 2004). An Aeris XRD analyzer by Malvern Panalytical was used, with the parameters set to 2*θ*° = 5–85. The analyzer was equipped with a cobalt anode and a Kα filter. The Rietveld method, specifically the software Profex, was used to evaluate the results (Doebelin & Kleeberg 2015). The amorphous phase was quantified using an internal standard, with 20% ZnO added to the sample.

## Results and discussion

### Factors affecting the MICP process and compactness of the samples (stage 1 testing)

Bacterial biomineralization plays a fundamental role in biogeochemical cycles and has significant technological and environmental applications that are greatly influenced by various factors and conditions (González-Muñoz et al. 2010). In our study, various factors such as pH adjustment, bacterial cell concentration and single or repeated addition of bacteria were tested on the success of the MICP treatment of WCF-G.

**Fig. 4:**
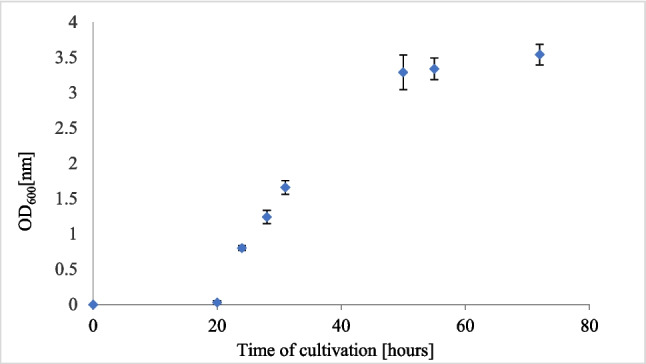
Growth curve of *Sporosarcina pasteurii* DSM 33

To ensure the highest ureolytic activity of *S. pasteurii* DSM 33 during the bacteria addition to WCF-G, the growth curve together with ureolytic activity was measured. The exponential phase was observed between 24 and 48 h of cultivation, followed by the entry of cells into the stationary phase (Fig. 4). Ureolytic activity was assessed concurrently with the growth curve using a phenol-hypochlorite ureolytic assay at six-time intervals. We found that the peak ureolytic activity occurred between 36 and 48 h, which corresponds to the late exponential phase. These results are consistent with previous studies (Sun et al. 2019a; Omoregie et al. 2020; Babakhani et al. 2021; Fang et al. 2021) and provide valuable insights for optimizing ureolytic activity in biocementation applications.

When examining the concentrations of bacterial cells (OD_600_ 2.5 and OD_600_ 5.0) with either single or repeated addition and the pH adjustment of WCF-G suspensions in stage 1, the concentration of bacterial cells turned out to be the crucial parameter for successful MICP. Similarly, Erdmann et al. (2022) determined the optimal bacterial concentration with OD_600_ of 1.6 for MICP of sandy waste materials. Interestingly, some studies using sand particles applied a significantly lower initial bacterial cell concentration, with an OD_600_ of 0.8–1.2 (Al Qabany et al. 2012; L. Chen et al. 2022). We chose the cell concentration for our experiment based on the studies of Abdel Gawwad et al. (2016), Chen et al. (2019) and Zhang et al. (2021), which showed that a bacterial culture with an OD_600_ of 2.4–2.5 resulted in greater calcite precipitation compared to samples with lower cell concentrations (OD_600_ of 0.5–1.0). In our experiment, all samples exhibited more compact WCF-G grains when the higher bacterial cell suspension (OD_600_ = 5.0) was applied. In these samples, a total of 68.0 ± 3.2% of the WCF-G particles aggregated in fractions larger than 15 mm and between 10 and 15 mm; in comparison, when a lower concentration of bacterial cells (OD_600_ = 2.5) was used, the proportion of these aggregated WCF-G-consolidated grains was only 48.0 ± 7.6%.

In addition, the compactness was found to be more pronounced in samples in which the initial pH of the suspension was adjusted to 6.8 ± 0.2. Typically, during calcite precipitation, the pH increases, and once it exceeds 10.0, the ureolytic activity decreases (Torres-Aravena et al. 2018). Therefore, WCF’s initial strongly alkaline environment (pH = 11–12) did not provided optimal conditions for MICP. This is in agreement with the studies of Gunjo Kim et al. (2018), Ghosh et al. (2019), Zhang et al. (2021), and Fu et al. (2022), who adjusted the pH to 6.5 ± 0.2. In contrast, Qiu et al. (2014) tested MICP with recycled concrete aggregate (RCA) with original pH 9.0–10.0 adjusted to 8.2, which is optimal for the growth of *S. pasteurii*. However, in this case, a lower cell concentration (~ 10^6^ cells/ml) was used to prevent a rapid increase in pH, which would halt calcite precipitation. Therefore, in our experiments, we adjusted the pH of the WCF-G suspension to 6.8 ± 0.2 to ensure CaCO_3_ precipitation. In this scenario, the medium flowing out from the containers had a pH between 9.5 and 11, indicating that the biocementation process had taken place.

When assessing the effects of the single or repeated addition of *S. pasteurii* DMS 33 (OD_600_ concentration of 5.0) at a consistent pH of the initial WCF-G suspension (6.8 ± 0.2), the consolidated WCF-G formed a compact structure that accounted for 61 to 87% of the entire sample. However, significant discrepancies were found in the presence of cracks and discontinuities within the samples. Based on the results (Table S3), most of the WCF-G samples did not form compact conglomerates. However, WCF-G samples with only single addition of bacterial suspension with OD_600_ = 5 and with pH adjustment produced stiff conglomerate with high stiffness. Other composites (with different tested parameters) resulted in poorly cemented conglomerates that disintegrated upon removal from the containers.

Based on these findings, in the subsequent experiment (stage 2), which tested the optimal duration of the MICP process, the following parameters were selected: adjustment of the initial pH of the WCF-G suspension to 6.8 ± 0.2, and the single addition of *S. pasteurii* DSM 33 (OD_600_ = 5.0).

### Effect of MICP duration (stage 2 testing)

**Fig. 5:**
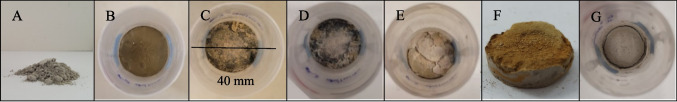
The WCF-H sample subjected to the biocementation process at stage 2: original sample (**A**), after 14 days (**B**), after 30 days (**C**), after 65 days (**D**), after 90 days of MICP (**E**), final compact composite of WCF-H after 90 days of MICP (**F**), and control (sterile) sample (**G**)

The tested experimental conditions were further verified on two types of WCFs (highway (WCF-H) and gutter (WCF-G)) differing in their physicochemical properties, and the objective was to analyze the effect of the length of biocementation (14, 30, 60, and 90 days) on the properties of the resulting composite materials. The formed composites, with six replicates for each variant, were characterized by the amount of CaCO_3_, mechanical properties and visual appearance, as shown in Fig. 5. Additionally, a more comprehensive analyses of the samples were performed using additional measurements, including SEM, EDS, and XRD.

### SEM analyses of specimens (stage 2 testing)

The CaCO_3_ crystals in the cemented WCF-G samples appeared as very fine formations, forming clusters and elongated columnar crystals. The crushed grains of the original concrete (including both aggregate and cement matrix) were sharp-edged and irregular.

**Fig. 6:**
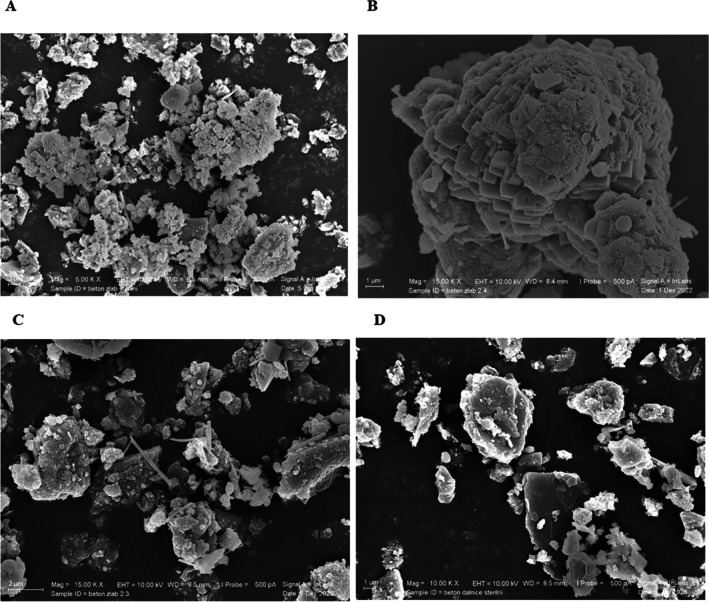
SEM images of CaCO_3_ crystals detected in MICP-cemented samples WCF-G (**A**) and WCF-H (**B**), and clusters of WCF grains covered with flaky CaCO_3_ crystals in sterile samples, WCF-G (**C**) and WCF-H (**D**). The images **A**–**D** were taken at magnifications of 15k × , 15k × , 5k × , and 10k × , respectively

Figure 6A shows the formation of CaCO_3_ of bacterial origin in clusters, ranging from small formations of up to 100 nm to elongated columnar crystals of 1.5 µm. In the sterile control samples, no such formations were observed. Recent research (Gu et al. 2022) reported that CaCO_3_ formations begin after 24 h and become more distinct after 48 h, and with repeated addition of the biocemention solution, the crystals continue to transform and form. For this reason, we also tested the factor of the duration of adding the biocemention solution and its effect on the morphology and size of the crystals. Finally, adding the biocemention solution for 14 days was insufficient to form CaCO_3_ crystals. Conversely, the duration of the addition of the biocementation solution (30, 60, and 90 days) had no effect on the structure, shape, or type of the crystals formed.

CaCO_3_ in the WCF-H sample occurred as small formations in crushed concrete (due to carbonation), as tiny flakes or scales (formed during solution application or sample crushing) (Qian et al. 2021; Gu et al. 2022), or as clusters of irregular formations (resulting from bacterial activity) (Bayati & Saadabadi 2021). The aggregate had sharp edges and parallel texture, while the cement matrix was both amorphous and sharp. The original concrete’s sharp-edged structure was acquired during the recycling process of crushing into fine powder.

Figure 6B shows bacterial-origin CaCO_3_ cluster formations ranging in size from several nanometers to 1 µm. On the other hand, sterile samples did not contain similar CaCO_3_ clusters or were only rarely visible and at very low concentrations. The analysis of sterile gutter and highway samples revealed leaf- or flake-like formations throughout (Fig. 6C, D) due to the crystallization of substances from the saline solution. The chemical composition of these areas confirmed that they consist of CaCO_3_.

### BSE-EDS measurements (stage 2 testing)

**Fig. 7:**
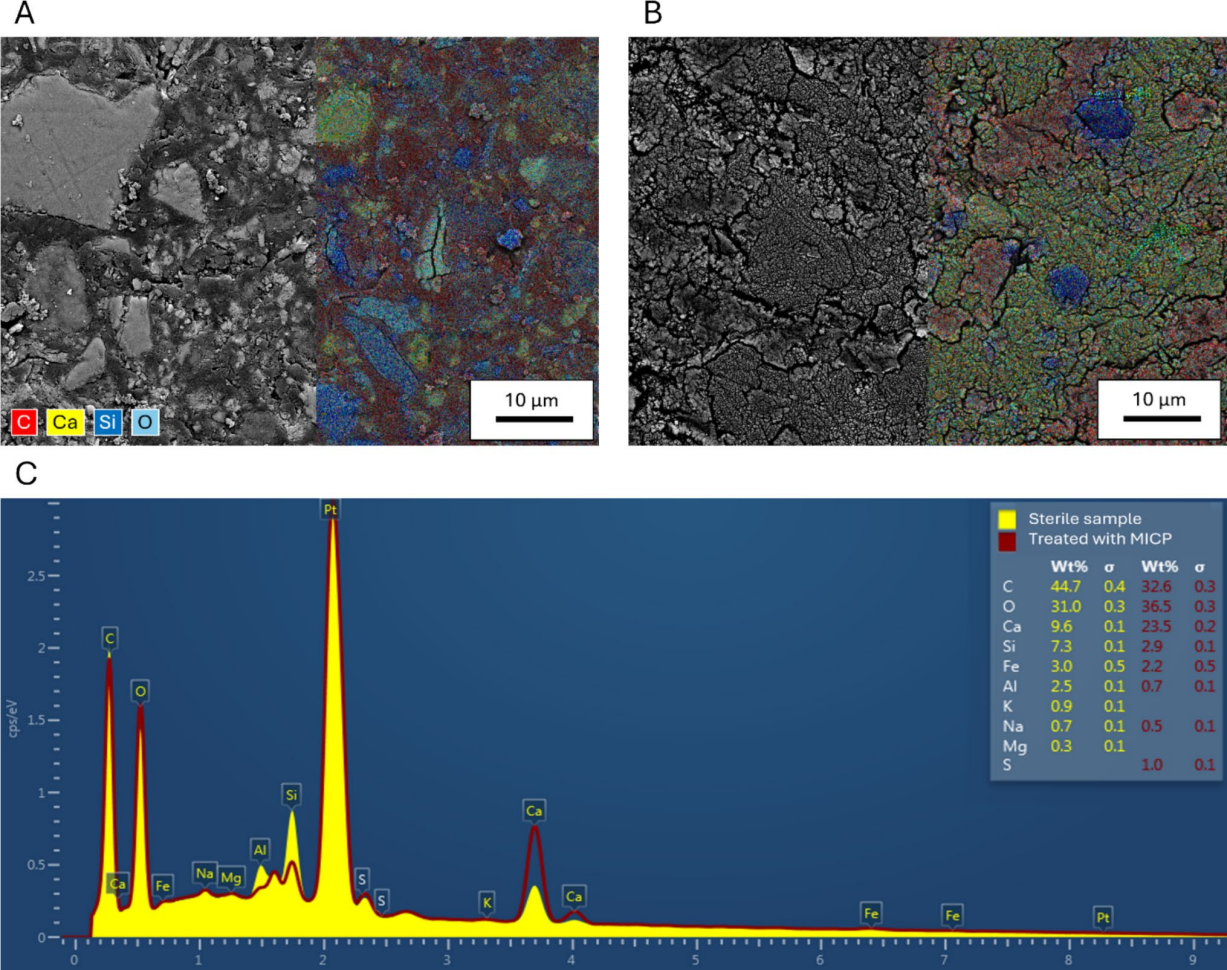
BSE images under 5k × magnification of the microstructure of sterile WCF-S-H90 (**A**) and biocemented WCF-H90 samples (**B**), complemented by color-coded map spectra of the different elements. Comparison of EDS spectra from the whole area of both images (**C**)

The presence of CaCO_3_ in the samples was verified by EDS analysis. The studied formations of the bacterial samples contained the elements carbon (C), calcium (Ca), and oxygen (O) in a certain ratio. From the ratio of these elements, it was concluded that they could be attributed to CaCO_3_ (Ghosh et al. 2019). Detailed information about the microstructure of the sample and the precipitated CaCO_3_ was obtained by SEM BSE microscopy. Figure 7A, B shows SEM images of the samples prepared by method 1, as described in the “Scanning electron microscope: microscopy imaging” section. A thin section for each sample was impregnated with epoxy resin and polished perfectly to form a flat surface, allowing EDS measurement to identify and quantify different phases. The SEM images are complemented by map spectra showing each element’s distribution. For better visualization, only the most represented elements have been highlighted in color: carbon in red, calcium in yellow, silicon in dark blue, and oxygen in light blue (O).

Figure 7A shows the microstructure of the sterile sample WCF-S-H90, demonstrating the high presence of siliceous aggregate (blue parts) and areas of cementitious matrix. The space between the grains was filled with carbon, representing the epoxy resin that filled the empty pores during sample preparation. Figure 7B shows the WCF-H90 sample treated with bacteria. This sample also contained silica aggregate and cementitious matrix. The voids and pores between the grains were largely filled with calcium carbonate (yellow–red areas). These CaCO_3_ formations were also observed in other studies (Tobler et al. 2018; Lin et al. 2020; Feng et al. 2021; Tang et al. 2023), even in a similar study to ours, dealing with sand biocementation (Iamchaturapatr et al. 2021). The biocemented samples contained mostly CaCO_3_ clusters with very few single crystals (Z. Wang et al. 2022b).

Other elements (iron, aluminum, sodium, magnesium, and sulfur) were not present in significant amounts, as documented by the energy spectrum generated from the whole area in Fig. 7C, comparing the spectra from the full image area. The energy spectrum of the sterile sample from the whole of Fig. 7A is shown in yellow, and the energy spectrum of the biocemented sample from the whole of Fig. 7B is shown in red.

EDS analysis of the sterile samples showed significantly lower concentrations of CaCO_3_, while the elements in the aggregate and cement matrix (Si, Fe, Al) were present in higher concentrations than in the MICP-treated samples. The trace amount of sulfur can be attributed to the origin of the WCF material and the presence of gypsum.

### XRD analysis: CaCO_3_ content (stage 2 testing)

**Fig. 8:**
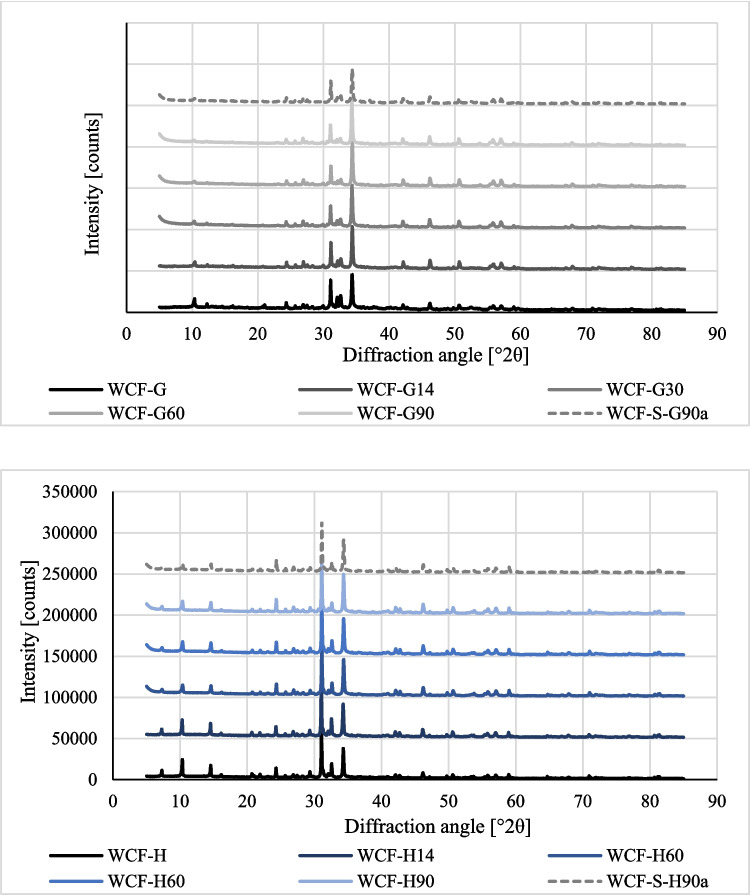
XRD diffractograms illustrating the shift from vaterite to more stable aragonite and predominant calcite forms over time in WCF-G and WCF-H samples. The comparison against untreated/sterile samples at 90 days (WCF-S-G90a and WCF-S-H90a) highlights increased calcite concentrations in treated samples within stage 2

CaCO_3_ may naturally occur in samples as aggregate, through carbonation, or as a result of bacterial activity (Bayati & Saadabadi 2021). The morphology of CaCO_3_ is divided, in addition to a rare amorphous structure (Scrivener et al. 2015), into three forms: calcite (the most stable form, which can form a wide variety of crystal shapes), aragonite (elongated, columnar crystals), and vaterite (the least stable form, characterized by oval formations) (Dhami et al. 2013; Ghosh et al. 2019; Omoregie et al. 2019; Šovljanski et al. 2021). The crystal form is influenced by many factors, such as the source of calcium, the kinetics of the precipitation process, and the composition of the biocement solution (Murugan et al. 2021). Previous studies (Mitchell and Ferris 2006; Heveran et al. 2019; Xu et al. 2020) described a transition from the less stable vaterite and aragonite phases to the more stable calcite phase during CaCO_3_ precipitation. Consistent with this trend, our findings demonstrate that calcite was the predominant form present in all biocemented samples (Fig. 8). As the MICP treatment progressed, the vaterite concentration decreased while the concentrations of the more stable aragonite and calcite crystals increased, as documented in Fig. 6, for both WCF-G and WCF-H samples. Furthermore, the untreated WCF samples and the sterile samples treated at 90 days exhibited similarly lower calcite concentrations.

**Fig. 9:**
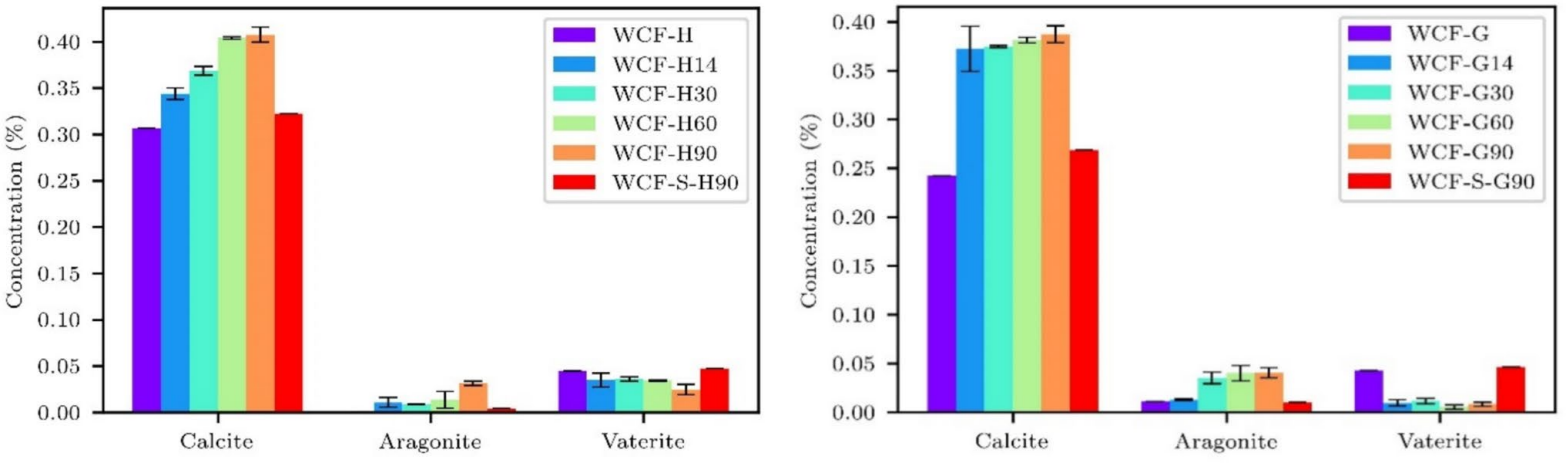
Concentration of CaCO_3_ crystals for different types of WCF materials and different treatments of the studied stage 2 samples based on XRD results

For a clearer representation of individual CaCO_3_ phases, see Fig. 9.

As for aggregate content, both sample types (WCF-G and WCF-H) contained similar minerals. The highway-derived samples revealed an aggregate composed of quartz, feldspar, and mica, as well as a probable presence of calcite (which appears in greater amounts than can be attributed solely to concrete carbonation). In the gutter-derived sample, a smaller amount of quartz and a reduced concentration of calcite were observed. Other studies that investigated WCF (Liu et al. 2022) and other alkaline materials, for example, alkali-activated slag mortar (Türker et al. 2016; Çelikten et al. 2019; Bayati and Saadabadi 2021), found that the aggregates contained similar proportions of quartz and feldspar, among other similarities. In all cases, the presence of quartz and feldspar is assumed to positively affect the precipitation of CaCO_3_ crystals and the effective filling of pores and microcracks in highly alkaline materials. Subsequent studies were focused on the impact of the size of WCF on final porosity in composite samples and their mechanical stiffness (Holeček et al. 2023).

### Evaluation of compactness and strength for samples within stage 2

**Fig. 10:**
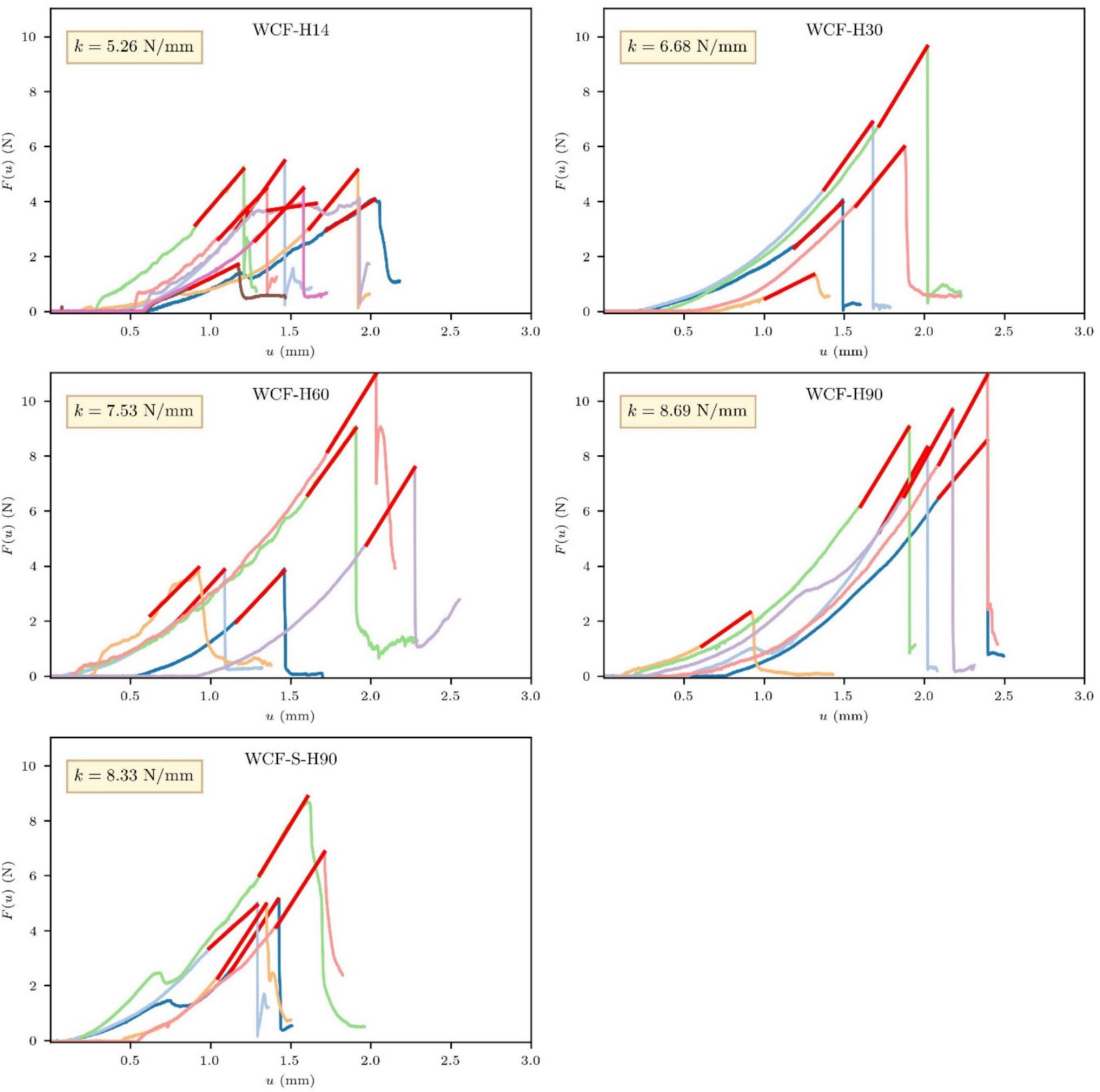
Load–displacement diagrams obtained during the indentation tests for the WCF-H samples

**Fig. 11:**
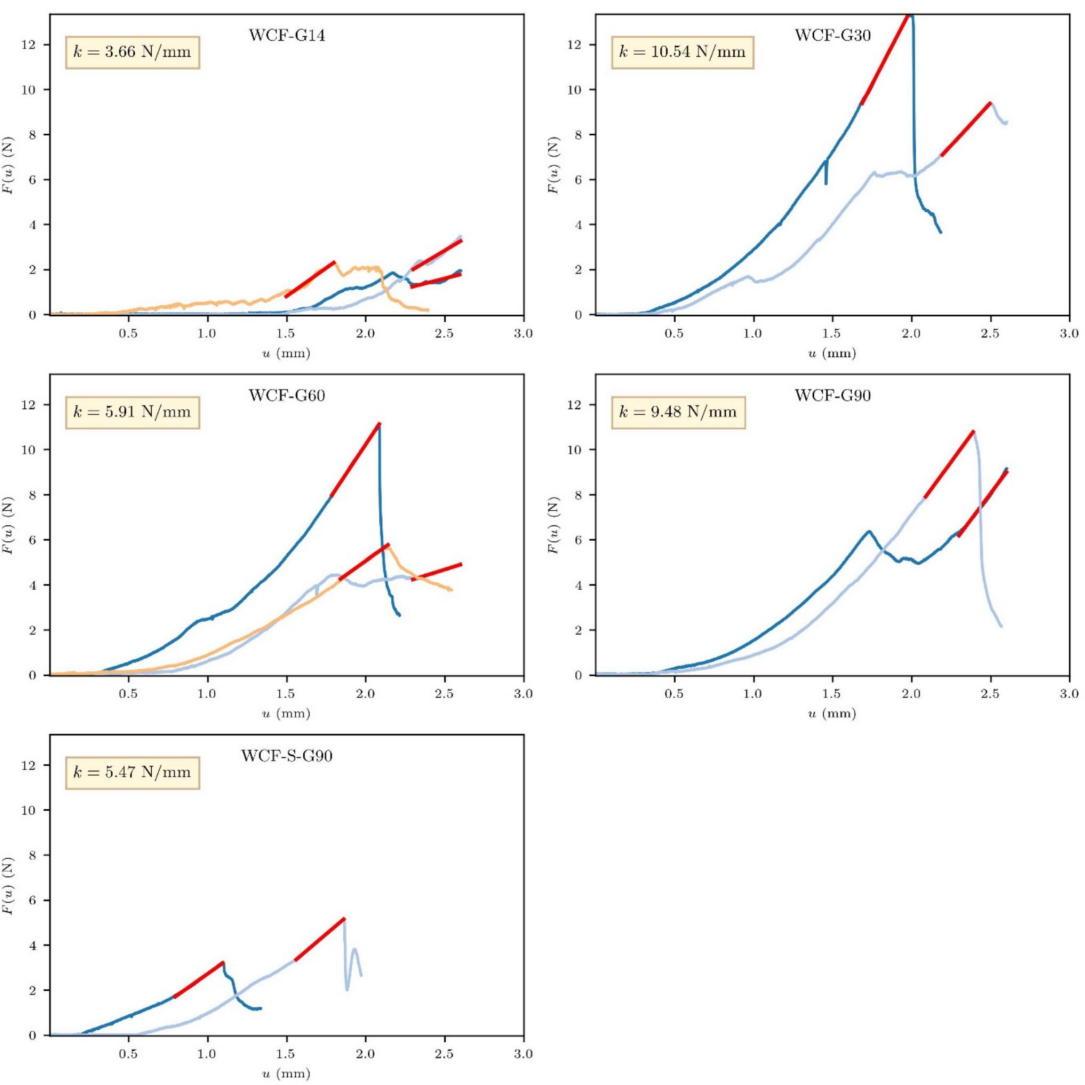
Load–displacement diagrams obtained during the indentation tests for the WCF-G samples

Given that the indentation method exceeds the microstructural scale, the indentation curves obtained reflect the bulk mechanical behavior of the material. For the conglomerates, elastic stiffness (*k*) was assessed as the tangent to the linear portion of the load–displacement (*F*(*u*)-*u*) curves (Figs. 10 and 11). Consistency was maintained by defining the linear interval for stiffness calculation as a uniform 0.4 mm displacement leading up to the peak load. This standardization ensures direct comparability of stiffness values across different samples.

This study hypothesizes that material stiffness is indicative of the strength of chemical bonds within the WCF conglomerates, given that these bonds influence the bulk mechanical properties of the material.

Most of the sterile WCF-G samples, or those subjected to MICP for a short duration (14 days), did not form compact conglomerates. This lack of compactness correlates with a low calcite concentration, as identified by XRD analysis (Table S4). Samples of WCF-G that underwent extended treatment and both sterile and MICP-treated WCF-H samples exposed to short treatment (14 days) generally resulted in poorly cemented conglomerates that disintegrated upon removal from the containers. However, longer treatment periods (30 to 90 days) for WCF-H samples produced compact conglomerates with significantly enhanced stiffness.

**Fig. 12:**
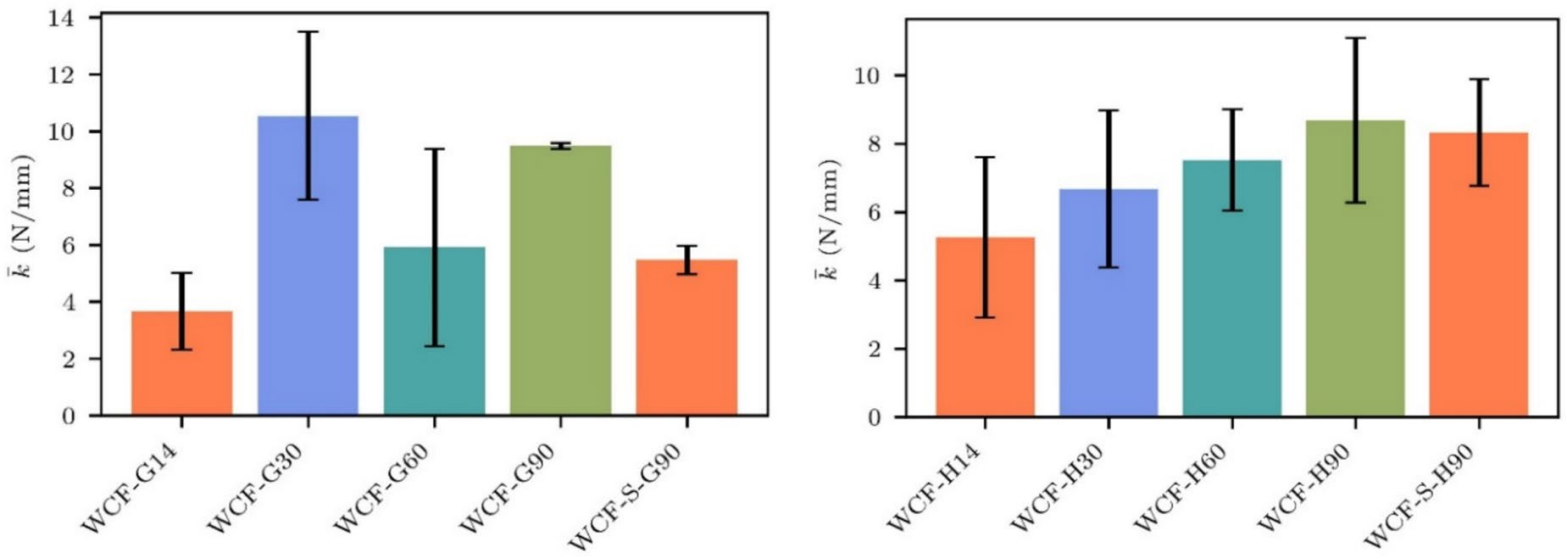
Indentation stiffness *k* evaluated for WCF-H samples (left) and WCF-G samples (H)

Interestingly, the sterile WCF-S-H90 and MICP-treated WCF-G30 samples that were sufficiently compact for indentation demonstrated relatively high stiffness values (Fig. 12). It is important to note, though, that the measured stiffness values showed considerable variability.

## Conclusion

Our study unveils a novel avenue for recycling disintegrated construction waste materials, extending promising applications to a broader spectrum of bulk construction waste and creating new opportunities to reduce the carbon footprint and support circular economy goals. In stage 1, we conducted initial parameter testing (pH adjustement, cell concentration and single or repeated addition of bacterial suspension) based on literature values to confirm that MICP processes, typically applied to soils and sands, could also be effective for WCF. This allowed us to refine the MICP treatment parameters for stage 2, where we focused on systematically evaluating the impacts of treatment duration. Our results demonstrate that the MICP strategy using ureolytic bacteria is indeed a viable approach for recycling WCF, contributing to a more sustainable option for concrete waste reuse. Specifically, we observed that time, pH, and varying concentrations of bacterial suspension significantly influence the consolidation and aggregation of individual grains into compact WCF samples. The biocemented samples contained a variety of crystal sizes, with crystals as large as 5 μm identified through SEM analysis, while XRD confirmed the presence of aragonite and calcite as the primary CaCO_3_ polymorphs formed during MICP, listed in ascending order of thermodynamic stability. Notably, the content of the less stable vaterite decreased slightly during MICP, transitioning into more stable forms in both types of WCF samples, originating from a prefabricated gutter and an old highway concrete layer. This suggests that the duration of MICP treatment influences CaCO_3_ polymorph distribution, as shown by the low concentrations of calcite and aragonite detected in untreated, sterile WCF samples. Mineral composition analysis of both types revealed similar aggregate contents; however, the highway-derived samples exhibited a higher calcite content with aggregates of quartz, feldspar, and mica, whereas the gutter-derived samples contained less quartz and a lower calcite concentration.

To improve the sustainability of this recycling process, future studies should focus on assessing economic and environmental feasibility through life cycle assessment. Utilizing agricultural or food industry waste as alternative nutrient sources for bacterial growth or calcite precipitation could help lower costs and increase affordability, given the relatively high expenses of the current approach. Other limitation of this study lies in the strength and stiffness of the resulting samples. Future research should thus conduct an in-depth evaluation of the mechanical properties of the composites, considering the physicochemical characteristics of the WCF. Another solution may be to use bacteria adapted to lower temperatures, which would also reduce the cost of the technology. Additionally, exploring strategies like reinforcing WCF materials with natural fibers or applying compression could enhance the performance and viability of this method. Ultimately, our research contributes to the growing body of knowledge on sustainable construction materials and highlights the potential of MICP as a viable approach to recycling construction waste.

## Supplementary information

Below is the link to the electronic supplementary material.

ESM 1 (DOCX 19.9 KB)

## Data Availability

The datasets used and/or analyzed during the current study are available from the corresponding author on request.

## Abbreviations

BSE: Backscattered electron
EDS: Energy-dispersive spectrometry
MICP: Microbially induced calcite precipitation
NB: Nutrient broth
OD: Optical density
PTFE: Polytetrafluorethylene
RCA: Recycled concrete aggregate
SEM: Scanning electron microscopy
TSA: Trypton soya agar
TSB: Trypton soya broth
WCF: Waste concrete fines
WCF-H: Waste concrete fines–highway
WCF-G: Waste concrete fines–gutter
XRD: X-ray diffraction
