# Supplementary material for: Application of Sporosarcina pasteurii for the biomineralization of calcite in the treatment of waste concrete fines
Source: Environ Sci Pollut Res Int. 2025 Feb 26;32(48):27989–8005. doi: 10.1007/s11356-025-36102-2 (PMC12696149; doi:10.1007/s11356-025-36102-2)
Supplement: Supplementary file 1 — (DOCX 19.9 KB) [file 11356_2025_36102_MOESM1_ESM.docx]

Table S1: Summary of prepared and tested specimens in stage 1.

| **Samples** | **DSM 33**  **OD_600_=5**  **[ml]** | **DSM 33**  **OD_600_=2.5**  **[ml]** | **pH adjusted to**  **6.8 ± 0.2 (1M HCl)**  **[ml]** | **MICP solution^a^**  **[ml]** |
| --- | --- | --- | --- | --- |
| WCF-G-01 | 10+10^b^ | - | - | 10 |
| WCF-G-02 | - | 10+10^b^ | - | 10 |
| WCF-G-03 | 10 | - | 2 | 10 |
| WCF-G-04 | 10+10^b^ | - | 2 | 10 |
| WCF-G-05 | - | 10+10^b^ | 2 | 10 |

*^a^* solutions were added consistently every 48 to 72 hours

^b^ repeated addition of bacterial suspension

Table S2: Summary of prepared and tested specimens in stage 2.

| **Samples** | **MICP treatment** | **DSM 33**  **OD_600_=5**  **[ml]** | **Saline***^b^*  **[ml]** | **MICP**  **(biocementation)**  **solution***^c^*  **[ml]** |
| --- | --- | --- | --- | --- |
| WCF-G14 | 14 days | 10 | - | 10 |
| WCF-G30 | 30 days | 10 | - | 10 |
| WCF-G60 | 60 days | 10 | - | 10 |
| WCF-G90 | 90 days | 10 | - | 10 |
| WCF-H14 | 14 days | 10 | - | 10 |
| WCF-H30 | 30 days | 10 | - | 10 |
| WCF-H60 | 60 days | 10 | - | 10 |
| WCF-H90 | 90 days | 10 |  | 10 |
| WCF-S-G90*^a^* | 90 days | - | 10 | - |
| WCF-S-H90*^a^* | 90 days | *-* | 10 | *-* |

*^a^* sterile samples

*^b, c^* solutions were added consistently every 48 to 72 hours

Table S3: Classification of samples based on their compactness.

| **Samples** | **1** | **2** | **3** |
| --- | --- | --- | --- |
| WCF-G-01 | Medium | Medium | Poor |
| WCF-G-02 | Poor | Poor | Poor |
| WCF-G-03 | Medium | Poor | High |
| WCF-G-04 | Poor | Poor | Poor |
| WCF-G-05 | Poor | Poor | Poor |

Table S4: Classification of samples based on their compactness.

| Samples | 1 | 2 | 3 | 4 | 5 | 6 |
| --- | --- | --- | --- | --- | --- | --- |
| WCF-S-G90 | Poor | Poor | Poor | Poor | Poor | Poor |
| WCF-G14 | Poor | Poor | Poor | Medium | Poor | Poor |
| WCF-G30 | Poor | Poor | Medium | Medium | Medium | Medium |
| WCF-G60 | Medium | Medium | Medium | Poor | Poor | Medium |
| WCF-G90 | Medium | Medium | Poor | Poor | Medium | Medium |
| WCF-S-H90 | Medium | Medium | Medium | Medium | Medium | Medium |
| WCF-H14 | High | High | High | Medium | Medium | Medium |
| WCF-H30 | High | High | High | Medium | Medium | High |
| WCF-H60 | High | Medium | High | High | High | High |
| WCF-H90 | Medium | High | High | High | High | High |
